# Supplementary material for: Cytomegalovirus-encoded immediate early 1 protein perturbs neural progenitor proliferation via interfering with host PML–DISC1 interaction
Source: J Biol Chem. 2026 Feb 6;302(3):111269. doi: 10.1016/j.jbc.2026.111269 (PMC12992096; doi:10.1016/j.jbc.2026.111269)
Supplement: Supporting information [file mmc1.pdf]

Figure S1

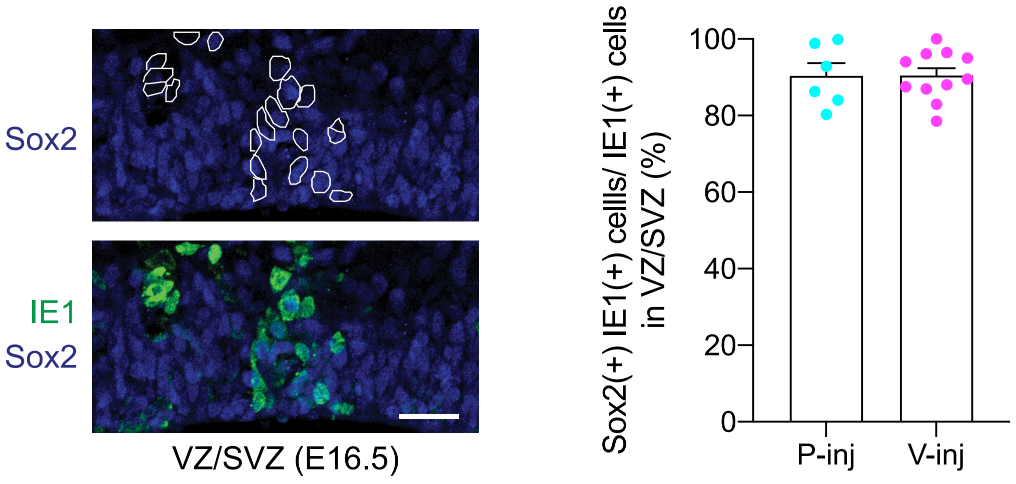

Sox2
IE1
Sox2
VZ/SVZ (E16.5)
Sox2(+) IE1(+) cells/ IE1(+) cells in VZ/SVZ (%)
100
80
60
40
20
0
P-inj
V-inj

Figure S1. IE1 highly localizes in Sox2-positive neural progenitor cells (NPCs) (related to Fig. 1).

Immunostaining for IE1 (green) and Sox2 (blue) in the ventricular zone / subventricular zone (VZ/SVZ). The white lines indicate the shapes of IE1 immunoreactivity. Scale bar, 50 µm. Graph shows percentage of Sox2-positive cells in IE1-positive cells (mean +/– s.e.m, n=6–11 images). There were no differences in the two models (p=0.9805; two-tailed Student's t-test).

Figure S2

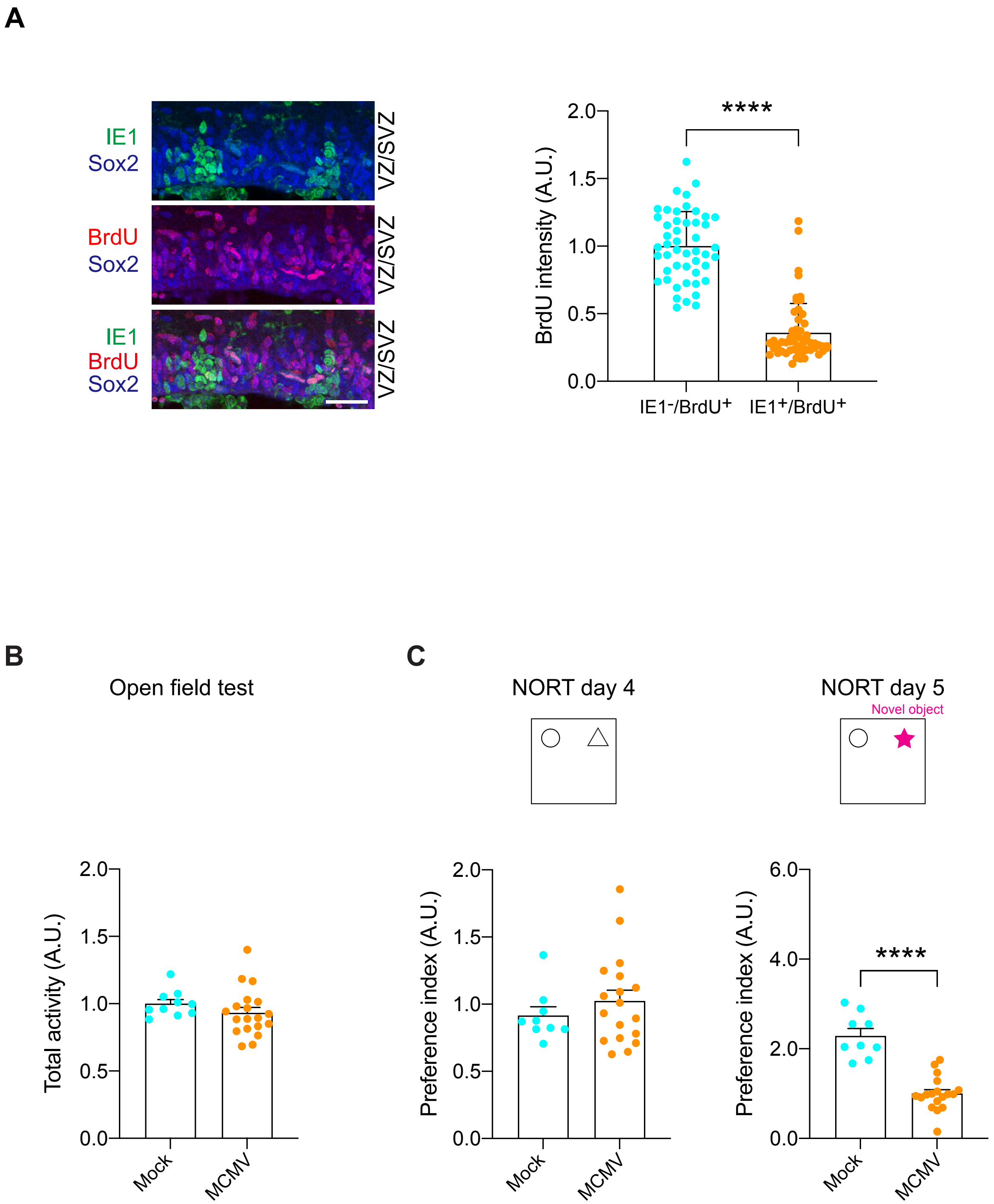
A
IE1
Sox2
BrdU
Sox2
IE1
BrdU
Sox2
VZ/SVZ
VZ/SVZ
VZ/SVZ
BrdU intensity (A.U.)
2.0
1.5
1.0
0.5
0.0
****
IE1-/BrdU+
IE1+/BrdU+
B
Open field test
Total activity (A.U.)
2.0
1.5
1.0
0.5
0.0
Mock
MCMV
C
NORT day 4
NORT day 5
Novel object
Preference index (A.U.)
2.0
1.5
1.0
0.5
0.0
Mock
MCMV
Preference index (A.U.)
6.0
4.0
2.0
0.0
****
Mock
MCMV

Figure S2. Intraventricular injection (V-inj) model shows impairment of neuronal development and a cognitive deficit (related to Fig. 2).

(A) Immunostaining for IE1 (green) and BrdU (red) shows a decrease in BrdU-labeled cells in MCMV-infected areas. IE1-positive nuclei rarely merged with BrdU-positive nuclei in the V-inj model. Scale bar, 50 μm. Graph shows BrdU incorporation in uninfected NPC (Sox2-positive but IE1-negative) and infected NPC (Sox2- and IE1- double positive). The ratio of BrdU- and Sox2- double positive nuclei to total Sox2-positive nuclei was significantly decreased in the infected area compared to the uninfected area. NPC, neural progenitor cell; Blue, Sox2; green, IE1; red, BrdU. Graph shows mean +/– s.e.m. (n=36-55 cells per group, ****$p<0.0001$; Mann-Whitney test).

(B) The total activity of mice infected with Mock and MCMV in the open field test. Total activity was not significantly different between groups. (Mock: n=10, MCMV: n=19; two-tailed Student's t-test).

(C) The results of the NORT in two groups (Mock and MCMV) in the ventricular injection (V-inj) model at 1 month after infection. Graphs indicate the preference index among the groups on day 4 and day 5 of testing. The preference index was calculated as the ratio of exploration time for novel object (or right object on day 4)/exploration time for both objects (day 5).

Graphs show mean +/– s.e.m. (Mock: n=9, MCMV: n=18, ****$p<0.0001$; two-tailed Student's t-test).

Figure S3

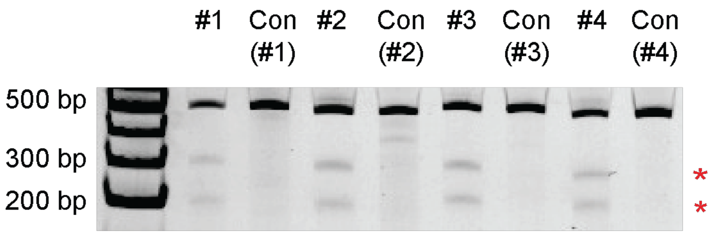

Figure S3. *In vitro* CRISPR validations (related to Fig. 3)

Viral IE1-targeting CRISPR/Cas9 cleaves the IE1 gene. The T7 Endonuclease I assay, which can detect genome cleavage caused by CRISPR/Cas9, shows that the IE1 genes in the MCMV genome are cleaved in MCMV-infected HT22 cells when co-transfected with the IE1-targeting CRISPR construct (IE1-CRISPR). Con, control. Red asterisks indicate cleavage products.

Figure S4

A

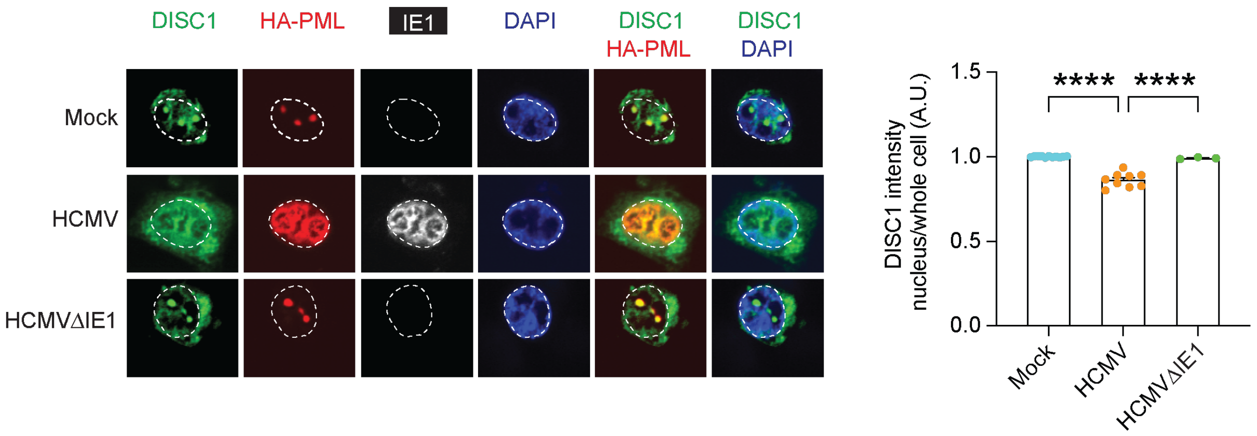

DISC1
HA-PML
IE1
DAPI
DISC1
HA-PML
DISC1
DAPI
Mock
HCMV
HCMVΔIE1
DISC1 intensity nucleus/whole cell (A.U.)
1.5
1.0
0.5
0.0
****
****
Mock
HCMV
HCMVΔIE1

B

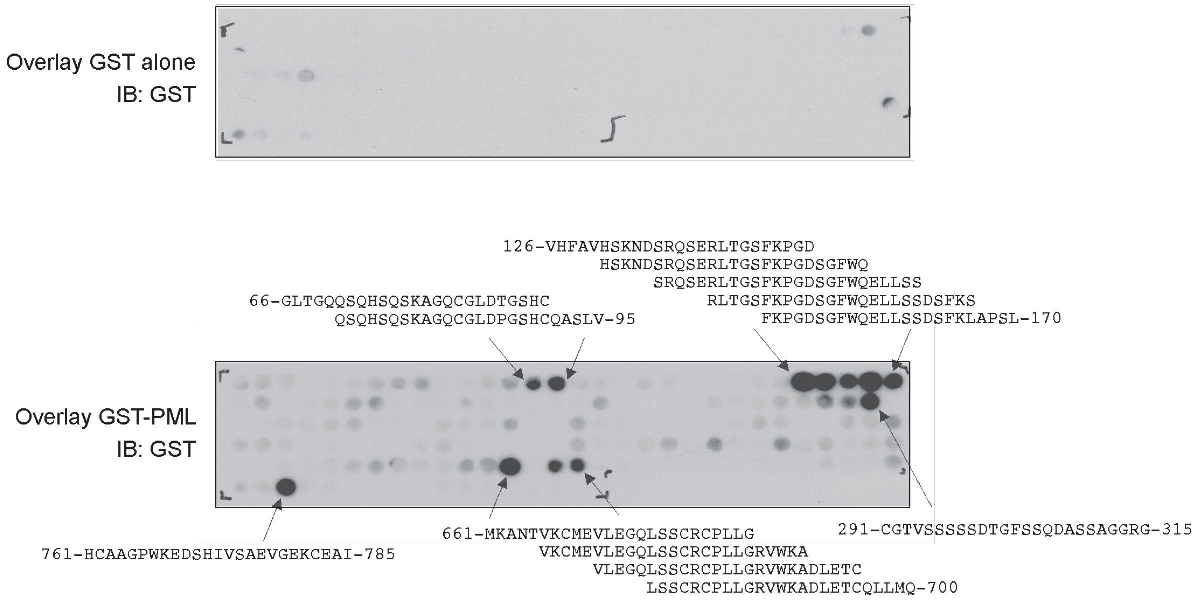

Overlay GST alone
IB: GST
126-VHFAVHSKNDSRQSERLTGSFKPGD
HSKNDSRQSERLTGSFKPGDSGFWQ
SRQSERLTGSFKPGDSGFWQELLSS
RLTGSFKPGDSGFWQELLSSDSFKS
FKPGDSGFWQELLSSDSFKLAPSL-170
66-GLTGQQSQHSQSKAGQCGLDTGSHC
QSQHSQSKAGQCGLDPGSHCQASLV-95
Overlay GST-PML
IB: GST
761-HCAAGPWKEDSHIVSAEVGEKCEAI-785
661-MKANTVKCMEVLEGQLSSCRCPLLG
VKCMEVLEGQLSSCRCPLLGRVWKA
VLEGQLSSCRCPLLGRVWKADLETC
LSSCRCPLLGRVWKADLETCQLLMQ-700
291-CGTVSSSSSDTGFSSQDASSAGGRG-315

Figure S4. *In vitro* validations (related to Fig. 4)

(A) Co-localization of DISC1-PML in HCMV-infected cells. Images show the nuclei of neuroblastoma cells. Infection with HCMV disturbed PML-DISC1 co-localization in human neuroblastoma. However, infection of HCMV lacking IE1 (HCMV ΔIE1) did not affect the PML-DISC1 co-localization. Green, DISC1; red, HA-PML; white, IE1; blue DAPI. Graph shows mean +/- s.e.m. (Mock: n=11, HCMV: n=9, HCMVΔIE1: n=3, Tukey's multiple comparison test *$p<0.05$, **$p<0.001$; one-way ANOVA: $F(2,20)=67.22$, $p<0.0001$].

(B) Pinpointing the PML binding domain on DISC1. DISC1 peptide arrays were probed for PML interaction sites. The dot blot shows interaction between GST-PML and a DISC1 peptide array.

Table S1A. Raw data for β-catenin assay (related to Fig. 5A)

Control RNAi		DISC1 RNAi		DISC1 RNAi + Wt DISC1		DISC1 RNAi + DISC1ΔPML	
Reporter (β-catenin)	Control (Renilla)	Reporter (β-catenin)	Control (Renilla)	Reporter (β-catenin)	Control (Renilla)	Reporter (β-catenin)	Control (Renilla)
1416	9525	607	9187	1805	9843	3755	20801
1545	10921	626	10272	1661	8353	3803	18961
1565	11336	558	10166	1474	8978	3658	18941
2964	9460	1555	9243	1369	8245	3987	32227
2940	11199	1484	9561	1542	8031	4076	33196
2926	11568	1381	9081	1278	7453	3918	36498
1485	16003	582	6928	2429	20319	1478	9319
1397	15718	539	7163	1990	19951	1771	8339
1594	16127	607	6899	2495	23736	1686	9026
2824	23710	942	11936	3133	11492	4342	16952
2750	27988	878	12877	3268	11632	4349	18117
3148	29397	894	12843	2819	11975	4075	18056
2663	10102	984	11940			3660	28758
2437	9581	1122	13283			3798	33442
2700	10805	1385	13429			3643	34985
3686	28595	1940	16533			1401	14609
3973	30204	2322	18816			1286	16486
3753	28287	2391	16772			1511	15311
3160	26545	743	7275				
4265	26386	833	7127				
3910	29516	861	7821				

Table S1B. Raw data for CBF1 assay (related to Fig. 5B)

Control RNAi		DISC1 RNAi		DISC1 RNAi + Wt DISC1		DISC1 RNAi + DISC1ΔPML	
Reporter (CBF1)	Control (Renilla)	Reporter (CBF1)	Control (Renilla)	Reporter (CBF1)	Control (Renilla)	Reporter (CBF1)	Control (Renilla)
1052	1583	11744	70078	3395	5068	2555	27048
1107	1547	11468	67183	2557	5676	2169	23719
1163	1616	12257	68235	2964	6282	2500	25398
6221	9683	832	4494	2280	3007	4474	15576
6621	9751	857	4304	2150	3305	4045	15090
5901	9567	889	4304	2121	3476	4581	17952
4562	8153	5839	44126	4681	7913	5625	22790
4758	7253	5758	40699	4094	9364	5166	24927
5039	6793	5908	40812	4101	9345	6378	23991
8883	16171	4979	34153	1610	2669	21688	100475
8327	18204	4649	40049	1669	3059	20003	93181
8372	19560	4995	37116	1770	2748	27808	92022
				15998	22791		
				14810	21700		
				16639	21781		
				6425	37426		
				6568	38454		
				7320	43031		
				3335	7693		
				3502	7708		
				3485	7767		

Relative Luminescence Units (RLU)

Table S2. Single guide RNA target sequences.

Target	Sequence	PAM sequence
IE1-CRISPR#1	5'- CGGCACGCTCATCTAGTGCG -3’	TGG
IE1-CRISPR#2	5'- AGATTAGTGGGCATGAAGTG -3’	TGG
IE1-CRISPR#3	5'- CACTAGATGAGCGTGCCGCA -3’	TGG
IE1-CRISPR#4	5'- GATGCGCTCGAAGATATCAT -3’	TGG

Table S3. T7 endonuclease I assay primer sequences.

Target	Primer
IE1-CRISPR#1	Fw: 5'- GATATCTTCGAGCGCATCGA -3’<br>Re: 5’- ACACACCCTGTGATATTGG -3’
IE1-CRISPR#2	Fw: 5'- CTGTTGTCCTGTAAGATTGC -3’<br>Re: 5’- CTTCCACCACTACCACATG -3’
IE1-CRISPR#3	Fw: 5'- TCGAAAGACAACGCAAGATG -3’<br>Re: 5’- GGTCTCTAGATGGTCTTTCATG -3’
IE1-CRISPR#4	Fw: 5'- CTCAGTTATTCACATCATGAC -3’<br>Re: 5’- TGTAACAGGGTGGATCATG -3’
